# Identification and validation of smoking-related genes in lung adenocarcinoma using an in vitro carcinogenesis model and bioinformatics analysis

**DOI:** 10.1186/s12967-020-02474-x

**Published:** 2020-08-14

**Authors:** Jin Wang, Tao Chen, Xiaofan Yu, Nan OUYang, Lirong Tan, Beibei Jia, Jian Tong, Jianxiang Li

**Affiliations:** 1 Department of Toxicology, School of Public Health, Medical College of Soochow University, Renai Road, Suzhou, 215123 China; 2 Jiangsu Key Laboratory of Preventive and Translational Medicine for Geriatric Diseases, Renai Road, Suzhou, 215123 China

**Keywords:** Cigarette smoke, Lung adenocarcinoma (LUAD), DNA methylation, prognosis, *GYPC*, *NME1*, *SLIT2*

## Abstract

**Background:**

Lung cancer is one of the most common carcinomas in the world, and lung adenocarcinoma (LUAD) is the most lethal and most common subtype of lung cancer. Cigarette smoking is the most leading risk factor of lung cancer, but it is still unclear how normal lung cells become cancerous in cigarette smokers. This study aims to identify potential smoking-related biomarkers associated with the progression and prognosis of LUAD, as well as their regulation mechanism using an in vitro carcinogenesis model and bioinformatics analysis.

**Results:**

Based on the integration analysis of four Gene Expression Omnibus (GEO) datasets and our mRNA sequencing analysis, 2 up-regulated and 11 down-regulated genes were identified in both S30 cells and LUAD. By analyzing the LUAD dataset in The Cancer Gene Analysis (TCGA) database, 3 of the 13 genes, viz., glycophorin C (*GYPC*), NME/NM23 nucleoside diphosphate kinase 1 (*NME1*) and slit guidance ligand 2 (*SLIT2*), were found to be significantly correlated with LUAD patients’ smoking history. The expression levels of *GYPC*, *NME1* and *SLIT2* in S30 cells and lung cancer cell lines were validated by quantitative PCR, immunofluorescence, and western blot assays. Besides, these three genes are associated with tumor invasion depth, and elevated expression of *NME1* was correlated with lymph node metastasis. The enrichment analysis suggested that these genes were highly correlated to tumorigenesis and metastasis-related biological processes and pathways. Moreover, the increased expression levels of *GYPC* and *SLIT2*, as well as decreased expression of *NME1* were associated with a favorable prognosis in LUAD patients. Furthermore, based on the multi-omics data in the TCGA database, these genes were found to be regulated by DNA methylation.

**Conclusion:**

In conclusion, our observations indicated that the differential expression of *GYPC*, *NME1* and *SLIT2* may be regulated by DNA methylation, and they are associated with cigarette smoke-induced LUAD, as well as serve as prognostic factors in LUAD patients.

## Background

Lung cancer is one of the most common carcinomas in the world. In 2018, the number of patients newly diagnosed with lung cancer across the globe was 2.09 million, and around 1.76 million patients will die from the disease [1]. Although early diagnosis and treatment of lung cancer have made significant progress, the 5-year relative overall survival (OS) is less than 20% [2]. Lung adenocarcinoma (LUAD) is the most common subtype of non-small cell lung cancer (NSCLC), and NSCLC accounts for approximately 85% of all lung cancer cases [3]. There is a significant and positive correlation between cigarette smoke and lung cancer, and the risk of developing lung cancer in smokers is nearly 10 times higher than that in non-smokers [4, 5]. Nonetheless, it is still unclear how normal lung cells become cancerous in cigarette smokers.

The development of high-throughput sequencing technology has made it possible to identify changes in single bases within the coding sequences of specific genes during lung tumorigenesis. There are plenty of publicly available cancer multi-omic data that we can obtain free from The Cancer Gene Atlas (TCGA; http://cancergenome.nih.gov/) and Gene Expression Omnibus (GEO, http://www.ncbi.nlm.nih.gov/geo/). A meticulous and thorough analysis of these data can identify genes and signaling pathways crucial to lung cancer, which will help for a better understanding of the mechanisms of cancer occurrence and development.

Based on the gene expression profiles, recent studies have identified several genes associated with lung cancer. Spira et al observed that *CYP*1*B*1, *NEK*2 and *CENPF* were significantly correlated with LUAD [6]. Liu et al suggested that *EPHA*4, *FGFR*2, and *EGFR* may be strongly associated with the development and progression of smoking-related LUAD [7]. Landi et al demonstrated that elevated mRNA levels of *NEK*2 and *TTK* have the potential to increase the risk of mortality from smoking-related LUAD [8]. Also, numerous genomic and transcriptional alterations in LUAD appeared to be associated with the patient’s smoking history [9]. However, there is still a shortage of reliable biomarkers for smoking-related LUAD.

**Fig. 1:**
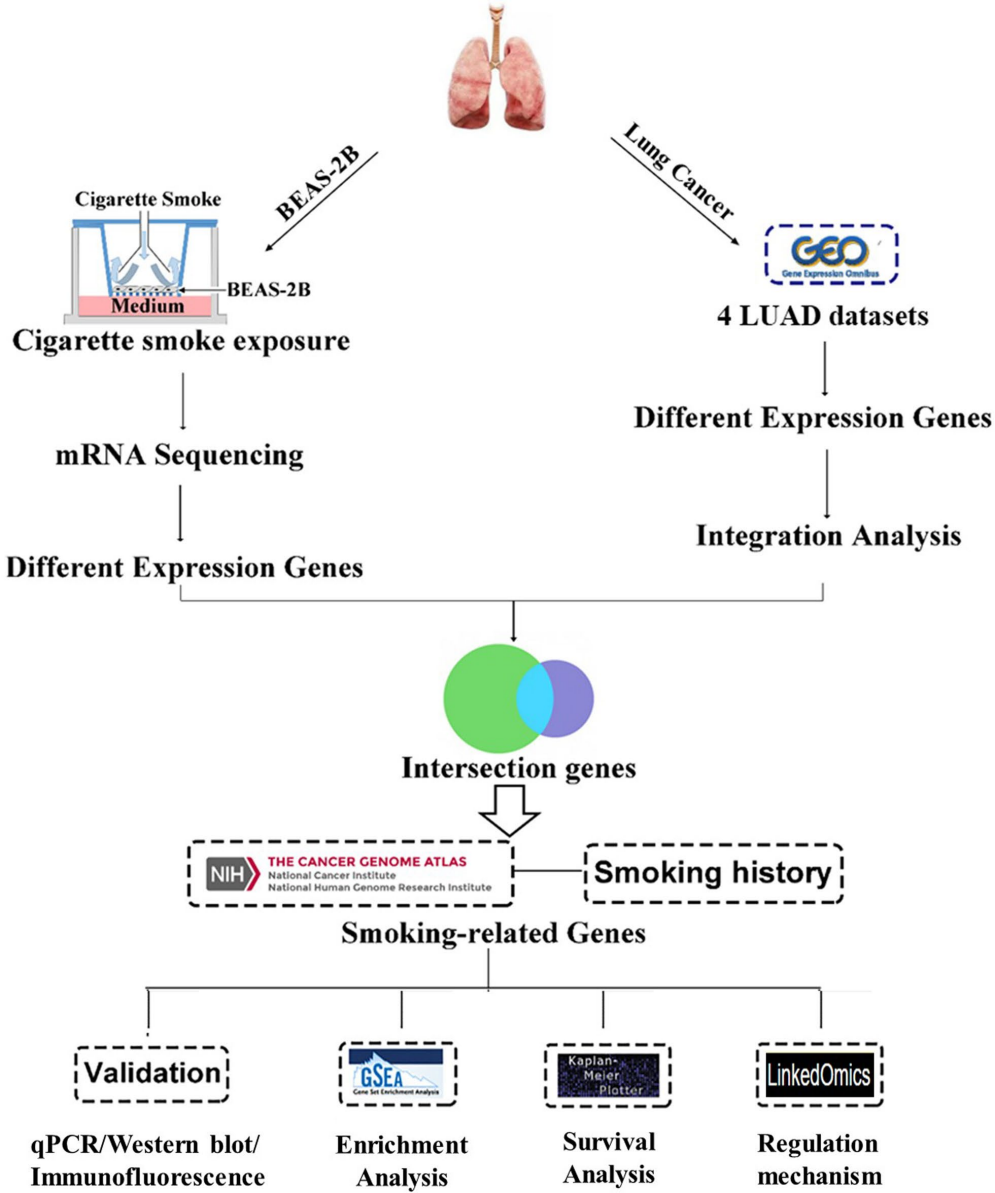
Workflow for identification of smoking-related genes in malignant transformation cells and LUAD. *LUAD* lung adenocarcinoma

In this study, we aimed to identify novel biomarkers for LUAD in smokers. The workflow of our study is presented in Fig. 1. An in vitro carcinogenesis model was established by exposing BEAS-2B cells to cigarette smoke continuously for 30 passages (S30). In the present study, candidate genes were obtained by integrative analysis of differentially expressed genes (DEGs) according to databases and our mRNA sequencing data. Among these, the smoking-related genes observed in S30 cells and LUAD were further validated by quantitative PCR (qPCR), immunofluorescence assays (IF), and western blotting (WB), and analyzed for a possible association with cancer-related pathways and prognosis. Furthermore, the multi-omics data in the TCGA database were used to explore the regulatory mechanisms of these three genes.

## Results

### Differentially expressed genes in S30 cells and GEO datasets

**Fig. 2:**
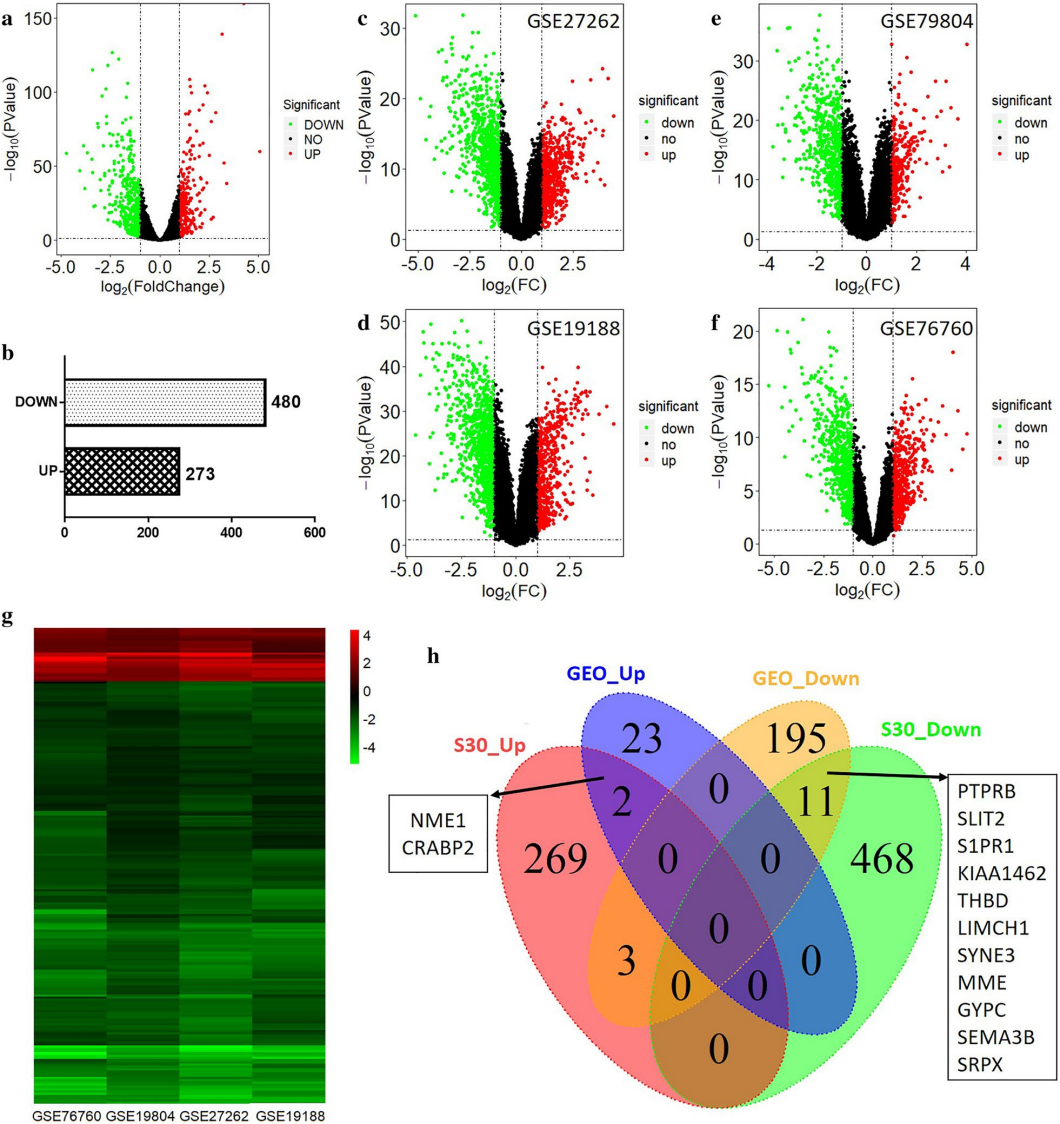
Identification of smoking-related genes in lung cancer. **a** A volcano plot was generated to visualize the distribution of DEGs. **b** Counts of upregulated or downregulated mRNAs. Volcano plots were generated to visualize the distribution of DEGs between LUAD tissues and adjacent normal tissues from different study cohorts, including GSE27262 (**c**), GSE19804 (**d**), GSE19188 (**e**) and GSE76760 (**f**). The X-axis of volcano plot indicates the fold change (FC, log-scaled), whereas the Y-axis shows the *p*-values (log-scaled). Each dot represents a different gene, and the red/green color of the dots categorizes the up-regulated/down-regulated genes under the filtering condition. **g** Heatmap of DEGs derived from integrated analysis. Each column represents one dataset and each row represents one gene; the gradual color ranged from green to red represents the changing process from down-regulation to up-regulation. **h** Venn diagram showing the overlap of identified DEGs from GEO datasets and cigarette smoke-induced malignant-transformation-cell model

Based on the high throughput analysis, a total of 753 differentially expressed genes (DEGs) were identified in cigarette smoke-induced transformed cells (S30) compared with unexposed BEAS-2B cells, including 273 up-regulated and 480 down-regulated genes (Fig. 2a, b). Besides, DEGs in LUAD tissues were screened out from four GEO datasets by differential expression analysis (Fig. 2c–f). Based on the integration analysis, 209 down-regulated genes and 25 up-regulated genes were identified in the GEO datasets (Fig. 2g and Additional file 1: Table S2). A total of 11 down-regulated and 2 up-regulated smoking-related genes were identified by taking the intersection of the DEGs extracted from S30 cells and GEO datasets (Fig. 2f).

### Identification of smoking-related genes in lung cancer

**Fig. 3:**
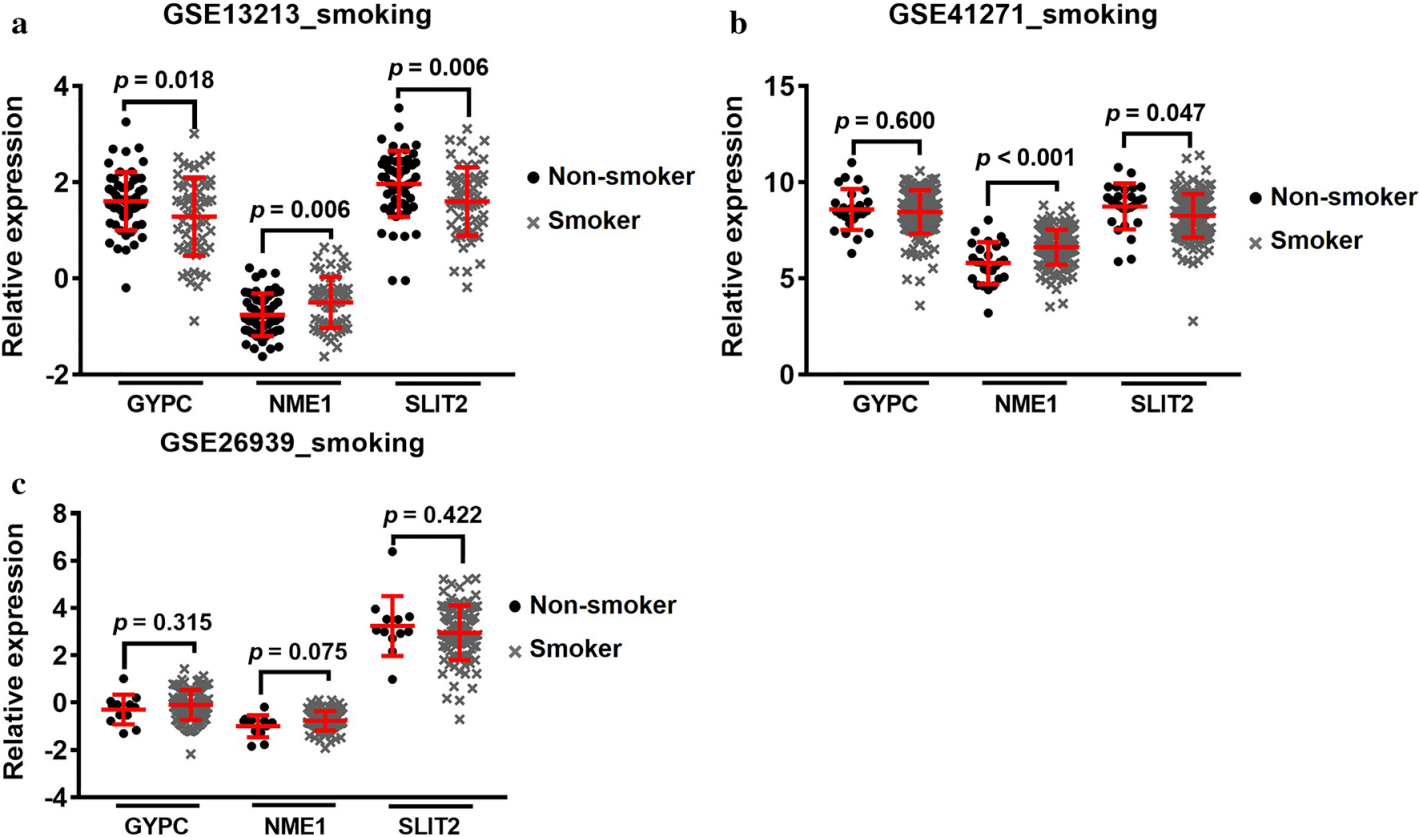
Validation of the association between mRNA expressions with smoking history in GEO datasets. **a** *GYPC*, *NME* and *SLIT2* expression levels were significantly different in smokers vs. non-smokers in GSE13213 dataset. **b** *NME* and *SLIT2* expression levels were significantly different in smokers vs. non-smokers in GSE41271 dataset. **b** The three genes expression levels showed no significantly different in smokers vs. non-smokers in GSE41271 dataset

Further analysis indicated that 7 of the 13 genes are associated with smoking history (*p* < 0.05) (Additional file 1: Table S3). Notably, the *NME1* expression level in current smokers and reformed smoker for $\le$15 years was significantly higher compared to life-long non-smokers (*p* < 0.01). Conversely, the expression levels of *SLIT2* and *GYPC* in current smokers were markedly lower than those in life-long non-smokers (*p* < 0.01) (Table 1). As illustrated in Fig. 3, these findings were further validated in two GEO datasets (GSE13213 and GSE41271) with a smoking history.

**Table 1:** Association of mRNA expression with LUAD patient tobacco smoking history in the TCGA database

Smoking history	Cases	*GYPC*	*NME1*	*SLIT2*
	(N)	(log2(RSEM+1))	(log2(RSEM+1))	(log2(RSEM+1))
1	75	8.64 ± 0.93	10.64 ± 0.98	7.85 ± 1.43
2	119	8.20 ± 1.00**	11.06 ± 0.70**	7.17 ± 1.53**
3	135	8.39 ± 0.82	10.62 ± 0.91^##^	7.69 ± 1.50^##^
4	168	8.47 ± 0.92^#^	10.99 ± 0.80**	7.54 ± 1.55^#^

1 = Lifelong Non-smokers (less than 100 cigarettes smoked in Lifetime), 2 = Current smokers (includes daily smokers and non-daily smokers or occasional smokers), 3 = Current reformed smokers for >15 years (greater than 15 years), 4 = Current reformed smokers for $\le$15 years (less than or equal to 15 years). Data represented are Mean ± SD, n depend on how many valid LUAD samples with corresponding factors. SD indicates standard deviation. *p < 0.05, versus Lifelong Non-smokers; **p < 0.01, versus Current smokers; ^#^*p* < 0.05, versus Lifelong Non-smokers; ^##^*p* < 0.01, versus Current smokers

### Validation of mRNA and protein expression in S30 cells and lung cancer cell lines

**Fig. 4:**
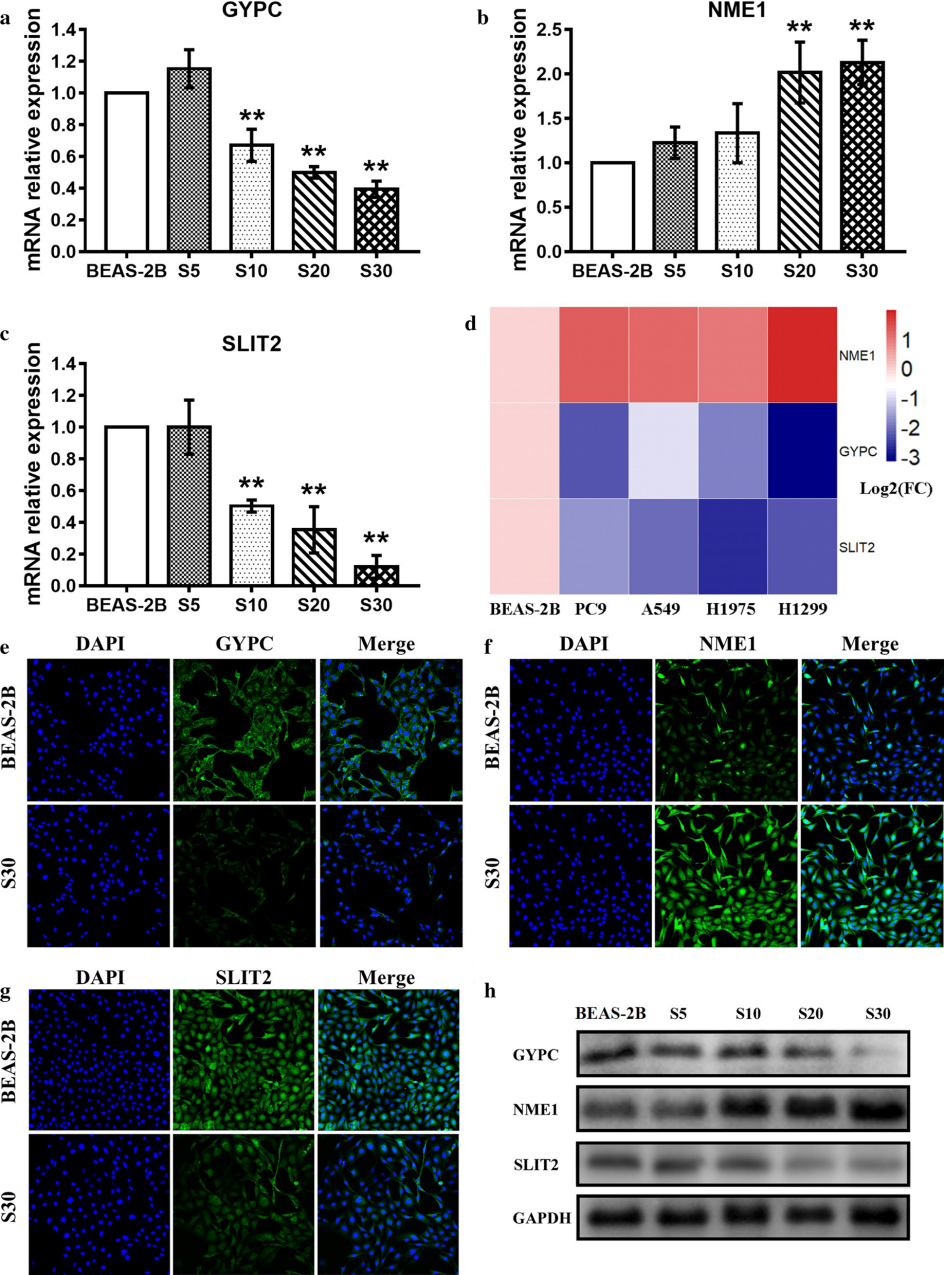
Validation of mRNA and protein expression levels of the three genes. The mRNA expression levels of *GYPC* (**a**), *NME1* (**b**) and *SLIT2* (**c**) in cigarette smoke-exposed cells. 2B, normal BEAS-2B cells serve as a control. S5 to S30, BEAS-2B cells exposed to cigarette smoke for different passages. **d** Heatmap of *GYPC*, *NME1* and *SLIT2* in BEAS-2B and four LUAD cell lines. Each column represents one cell line, and each row represents one gene; the gradual color ranged from blue to red represents the changing process from down-regulation to up-regulation. **e** Immunofluorescence staining of GYPC. **f** Immunofluorescence staining of NME1. **g** Immunofluorescence staining of SLIT2. **h** Western blotting results. S5 to S30, BEAS-2B cells exposed to cigarette smoke for different passages.*p < 0.05, versus normal BEAS-2B cells; **p < 0.01, versus normal BEAS-2B cells

The mRNA expression levels of *GYPC* and *SLIT2* were found to be dependent on smoke-exposure time and were significantly down-regulated in S30 cells (Fig. 4a, c). On the other hand, the NME1 expression level was significantly up-regulated in S30 cells (Fig. 4b). Compared with normal BEAS-2B cells, the expression levels of *GYPC* and *SLIT2* in four human lung adenocarcinoma cell lines (PC9, A549, H1975 and H1299) were up-regulated, while *NME1* expression was down-regulated (Fig. 4d). The protein expression levels of GYPC, NME1 and SLIT2 were further validated. Immunofluorescence staining showed that NME1 protein expression was increased in S30 cells compared with normal BEAS-2B cells, while GYPC and SLIT2 expression was decreased (Fig. 4e–g). Western Blot analysis further confirmed the downregulation of GYPC and SLIT2 and the upregulation of NME1in cigarette smoke-exposed S30 cells compared with unexposed BEAS-2B cells (Fig. 4h).

### Association of mRNA expression with pathological characteristics

We further investigated the association between the expression levels of the three genes (*NME1*, *SLIT2* and *GYPC*) and pathological features (Table 2). For invasion depth, the expression levels of *GYPC* and *SLIT2* were significantly decreased in LUAD tissues at the T2 stage compared with T1 stage tissues, while the expression level of *NME1* significantly increased. When lymph node metastasis was considered, the mRNA expression level of *NME1* was greater in N1 and N2 vs. N0. Besides, the mRNA expression level of *NME1* was upregulated in TNM stage III vs. stage I. It is worth mentioning that the expression levels of *GYPC* and *NME1* were significantly different between males and females, and *NME1* and *SLIT2* were substantially different between elder patients ($\ge$60 years old) and patients aged less than 60 years old.

**Table 2:** Association of mRNA expression levels of *GYPC*, *NME1* and *SLIT2* with the pathological features

Factor	Case	*GYPC*	*NME1*	*SLIT2*
Gender				
Male	238	8.31 ± 0.91	10.95 ± 0.86	7.44 ± 1.57
Female	277	8.52 ± 0.94*	10.80 ± 0.84*	7.63 ± 1.47
Age				
< 60	136	8.37 ± 0.99	11.00 ± 0.78	7.32 ± 1.61
$\ge$60	360	8.45 ± 0.91	10.82 ± 0.88*	7.63 ± 1.48*
Invasion depth				
T1	169	8.63 ± 0.77	10.67 ± 0.88	7.89 ± 1.31
T2	277	8.32 ± 0.99**	10.96 ± 0.76**	7.35 ± 1.57**
T3	47	8.38 ± 0.92	10.97 ± 1.11	7.35 ± 1.69*
T4	19	7.99 ± 0.87**	11.07 ± 0.98	7.34 ± 1.29
Lymph node metastasis				
N0	331	8.43 ± 0.95	10.78 ± 0.85	7.58 ± 1.55
N1	96	8.43 ± 0.89	11.04 ± 0.85**	7.34 ± 1.46
N2	74	8.27 ± 0.85	11.10 ± 0.81**	7.47 ± 1.44
N3	2	8.46 ± 0.19	11.04 ± 1.07	6.76 ± 0.46
Distant metastasis				
M0	346	8.43 ± 0.92	10.95 ± 0.85	7.50 ± 1.48
M1	25	8.06 ± 0.88	10.96 ± 0.91	7.66 ± 1.70
TNM stage				
I	275	8.46 ± 0.92	7.60 ± 1.49	10.78 ± 0.82
II	122	8.47 ± 0.99	7.47 ± 1.59	10.87 ± 0.85
III	84	8.30 ± 0.83	7.36 ± 1.41**	11.12 ± 0.89
IV	26	8.17 ± 1.02	7.78 ± 1.78	10.92 ± 0.91

Note: The data was presented as in log$_{2}$(x+1) transformed RSEM normalized count. LUAD, lung adenocarcinoma; TCGA: The Cancer Gene Atlas; TNM: tumor-node-metastasis; stage RSEM: RNA seq by expectation-maximization. * *p*<0.05, versus the first group of the corresponding feature. ** *p*<0.01, versus the first group of the corresponding feature

### Gene Ontology enrichment analysis

**Fig. 5:**
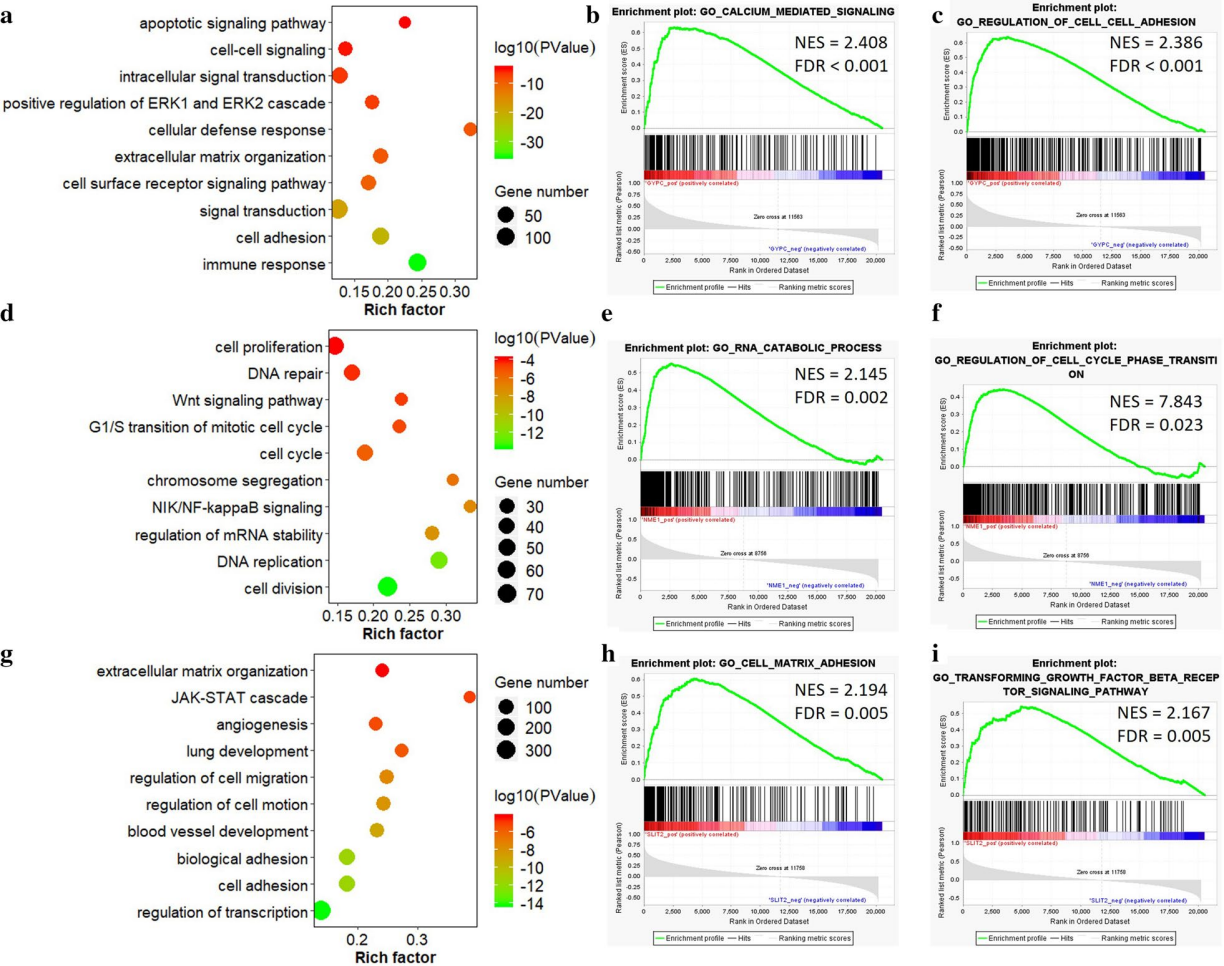
The GO enrichment results of GYPC, NME1 and SLIT2. **a**The bubble chart shows the GO terms related to *GYPC*; GSEA used to validate the gene signatures of *GYPC*, including (**b**) calcium mediated signaling and **c** regulation of cell-cell adhesion. **d** The bubble chart shows the GO terms related to *NME1*; GSEA used to validate the gene signatures of *NME1*, including **e** RNA catabolic process and **f** regulation of cell cycle phase transition. **g** The bubble chart shows the GO terms related to *SLIT2*; GSEA used to validate the gene signatures of *SLIT2*, including (H) cell matrix adhesion and **i** TGF-$\beta$ receptor signaling pathway

Based on the UALCAN online tool, a total of 1182, 1771 and 1822 genes significantly correlated with *GYPC*, *NME1* and *SLIT2* were extracted respectively. Gene ontology enrichment analysis was performed to demonstrate the potential biological functions of these related genes using DAVID. The results showed that these three genes were functionally associated with several critical biological processes. For *GYPC*, the genes co-expressed with it were remarkably enriched in apoptotic signaling pathway and extracellular matrix organization, as well as cell adhesion Fig. 5a). When *NME1* was considered, the related genes were found to be enriched in cell proliferation, DNA repair and cell cycle, as well as Wnt signaling pathway Fig. 5d). Besides, the genes correlated with *SLIT2* were significantly associated with extracellular matrix organization, JAK-STAT cascade, and cell adhesion Fig. 5g). In addition, GSEA enrichment analysis confirmed the three genes signatures, including calcium mediated signaling and regulation of cell-cell adhesion for *GYPC* Fig. 5b, c), RNA catabolic process and regulation of cell cycle phase transition for *NME1* Fig. 5e, f), as well as cell matrix adhesion and TGF-$\beta$ receptor signaling pathway for *SLIT2* Fig. 5h, i).

### KEGG pathway enrichment analysis

**Fig. 6:**
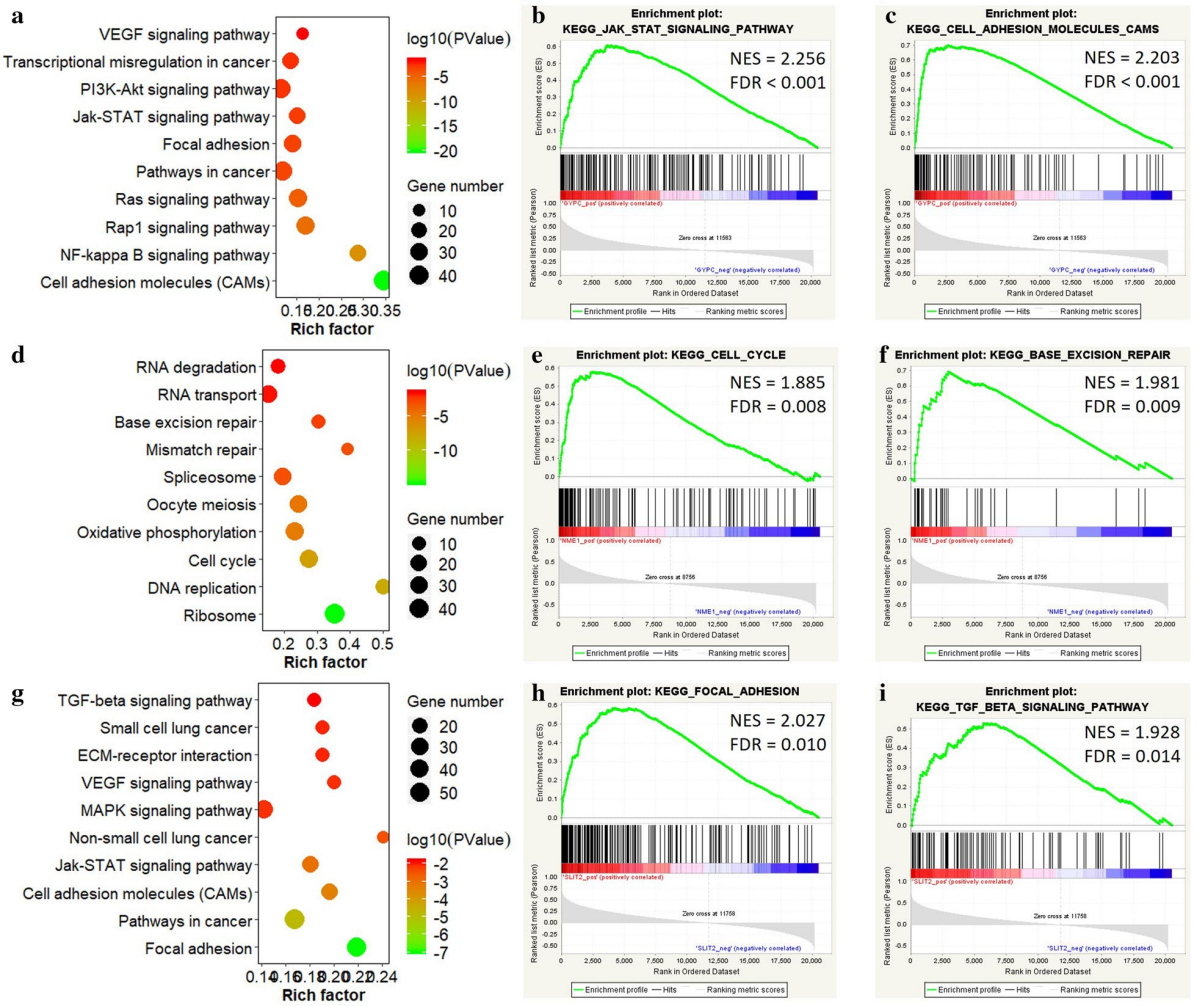
The KEGG enrichment results of GYPC, NME1 and SLIT2. **a**The bubble chart shows the KEGG pathways related to *GYPC*; GSEA used to validate the gene signatures of *GYPC*, including **b** JAK-STAT signaling pathway and **c** cell molecular adhesion. **d** The bubble chart shows the KEGG pathways related to *NME1*; GSEA used to validate the gene signatures of *NME1*, including **e** cell cycle and **f** base excision repair. **g** The bubble chart shows the GO terms related to *SLIT2*; GSEA used to validate the gene signatures of *SLIT2*, including **h** focal adhesion and **i** TGF-$\beta$ signaling pathway

To better illustrate the functional role of these three genes in lung cancer, KEGG pathway analysis was performed for the related genes using DAVID online tool. The results showed that these three genes were involved in multiple pathways in lung cancer. When considering GYPC, the related genes were involved in JAK-STAT, PI3K-Akt, and Ras/Rap1 signaling pathways, as well as focal adhesion and cell adhesion molecules (Fig. 6a). Besides, genes related to NME1 were found to be enriched in base excision repair, mismatch repair, and cell cycle (Fig. 6d). Similarly, the SLIT2 related genes were significantly associated with TGF-$\beta$ receptor, VEGF, MAPK and JAK-STAT signaling pathways, as well as lung small lung cancer and focal adhesion (Fig. 6g). The further GSEA enrichment confirmed these pathway signatures of GYPC (Fig. 6b, c), NME1 (Fig. 6e, f), and SLIT2 (Fig. 6h, i).

### Survival analysis

**Fig. 7:**
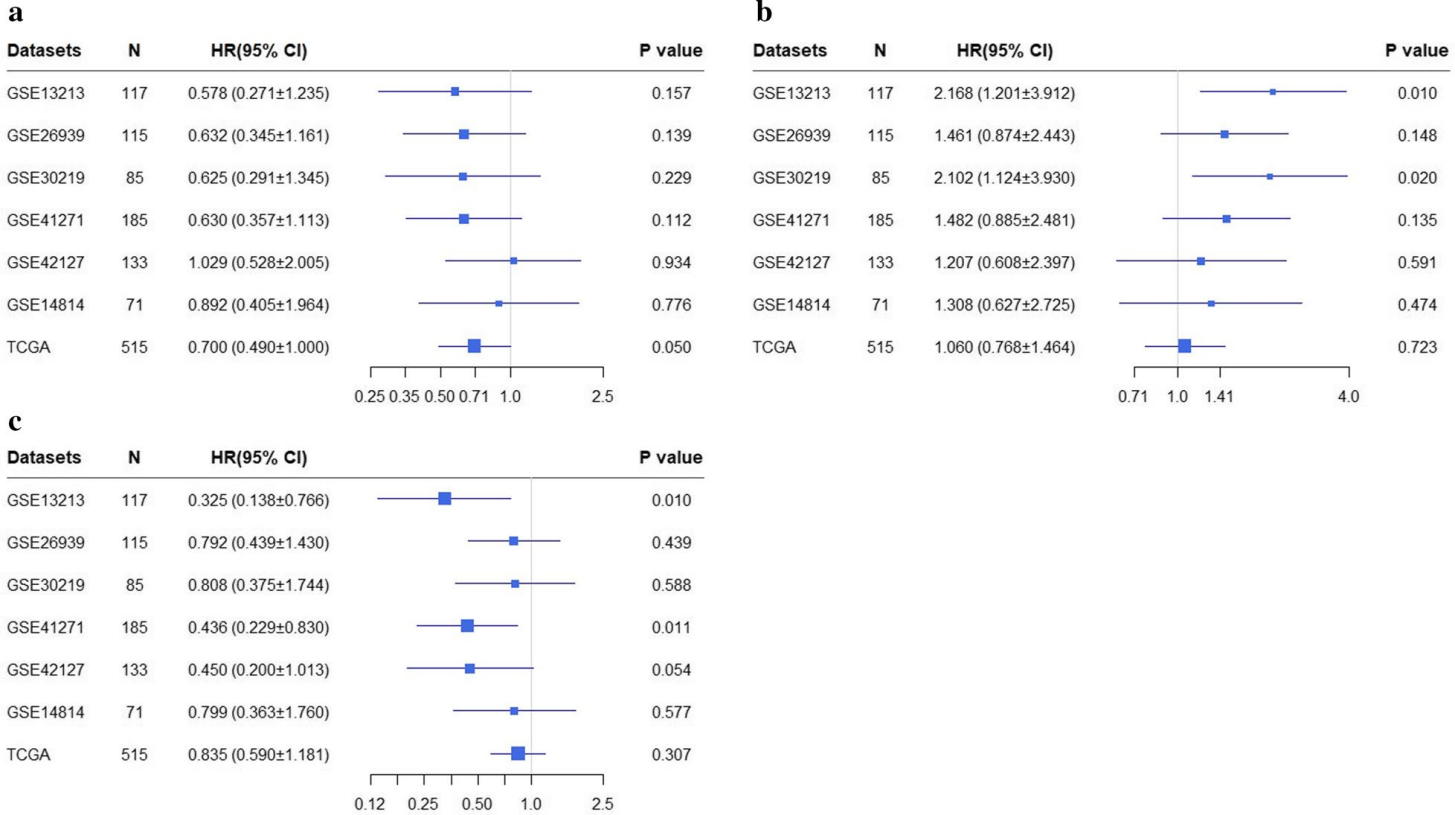
Forest plots based on univariate survival analysis in patients with LUAD. Univariate COX results of *GYPC* (**a**), *NME1* (**b**) and *SLIT2* (**c**) in patients with LUAD. *LUAD* lung adenocarcinoma, *HR* hazard ratio, *CI* confidence interval, *TCGA* The Cancer Genome Atlas

**Fig. 8:**
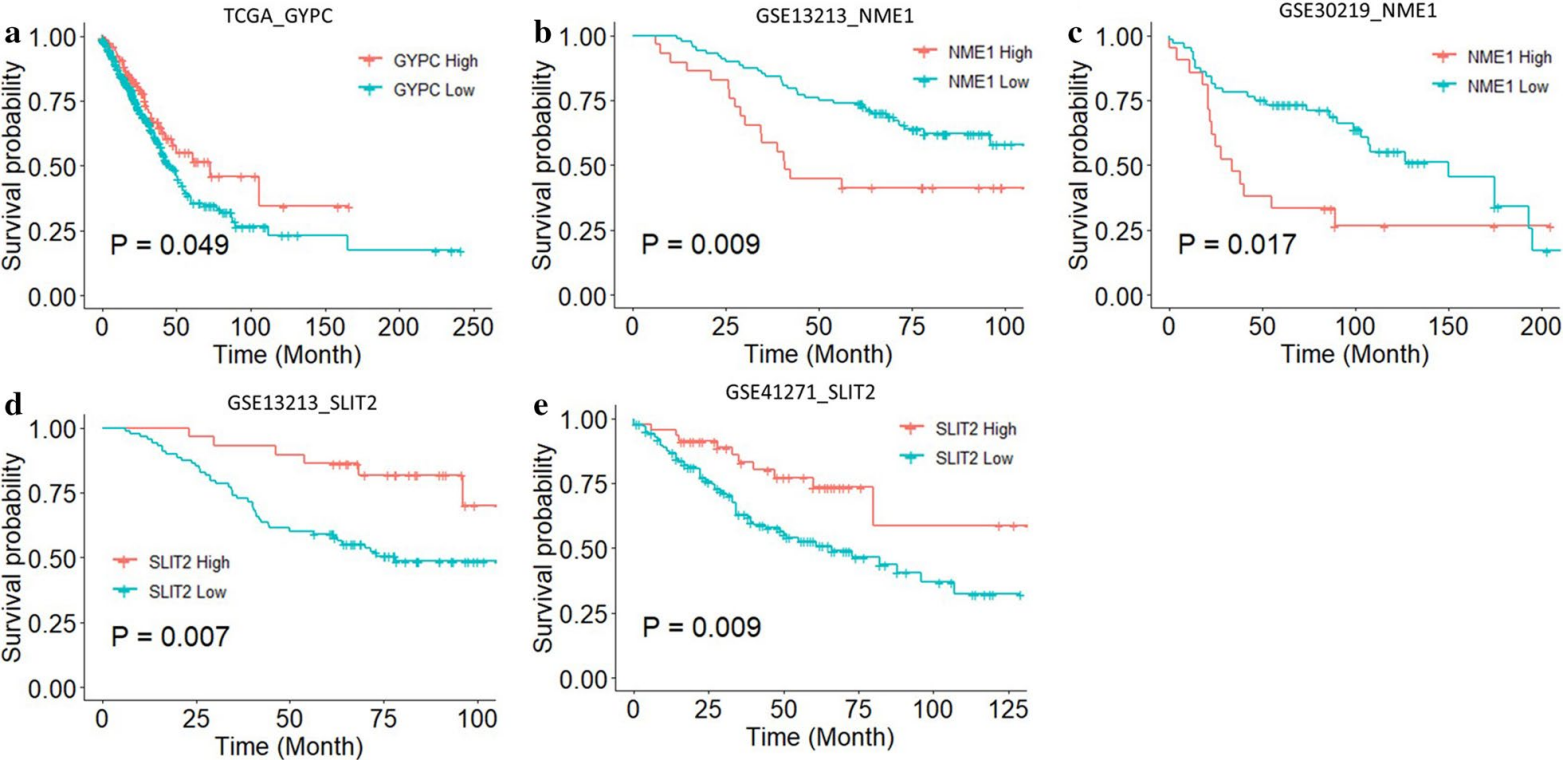
Kaplan–Meier survival analysis of LUAD patients by GYPC, NME1 and SLIT2 expression in different datasets. **a** Kaplan–Meier survival curve of overall survival (OS) based on *GYPC* expression in TCGA-LUAD dataset; **b** Kaplan–Meier survival curve of OS based on *NME1* expression in GSE13213 dataset; **c** Kaplan–Meier survival curve of OS based on *NME1* expression in GSE30219 dataset; **d** Kaplan–Meier survival curve of OS based on *SLIT2* expression in GSE13213 dataset; **e** Kaplan–Meier survival curve of OS based on *SLIT2* expression in GSE41271 dataset

A total of 7 data cohorts, including 1221 LUAD patients, were used to establish univariate Cox models. The univariate Cox model analyses of TCGA dataset suggested that higher *GYPC* expression has a favorite prognosis (HR < 1, *p* < 0.05) (Fig. 7a); The analysis in GSE13213 and GSE30219 datasets revealed that higher *NME1* expression was a risk factor for LUAD patients prognosis (HR < 1, *p* < 0.05) (Fig. 7b). In addition, the analysis in GSE13213 and GSE41271 indicated that increased SLIT2 expression is associated with a better prognosis (HR < 1, *p* < 0.05) (Fig. 7c). The KM survival analysis is consistent with the univariate Cox analysis (Fig. 8).

### Gene dysregulation is mediated by methylation and gene amplification in lung cancer

**Fig. 9:**
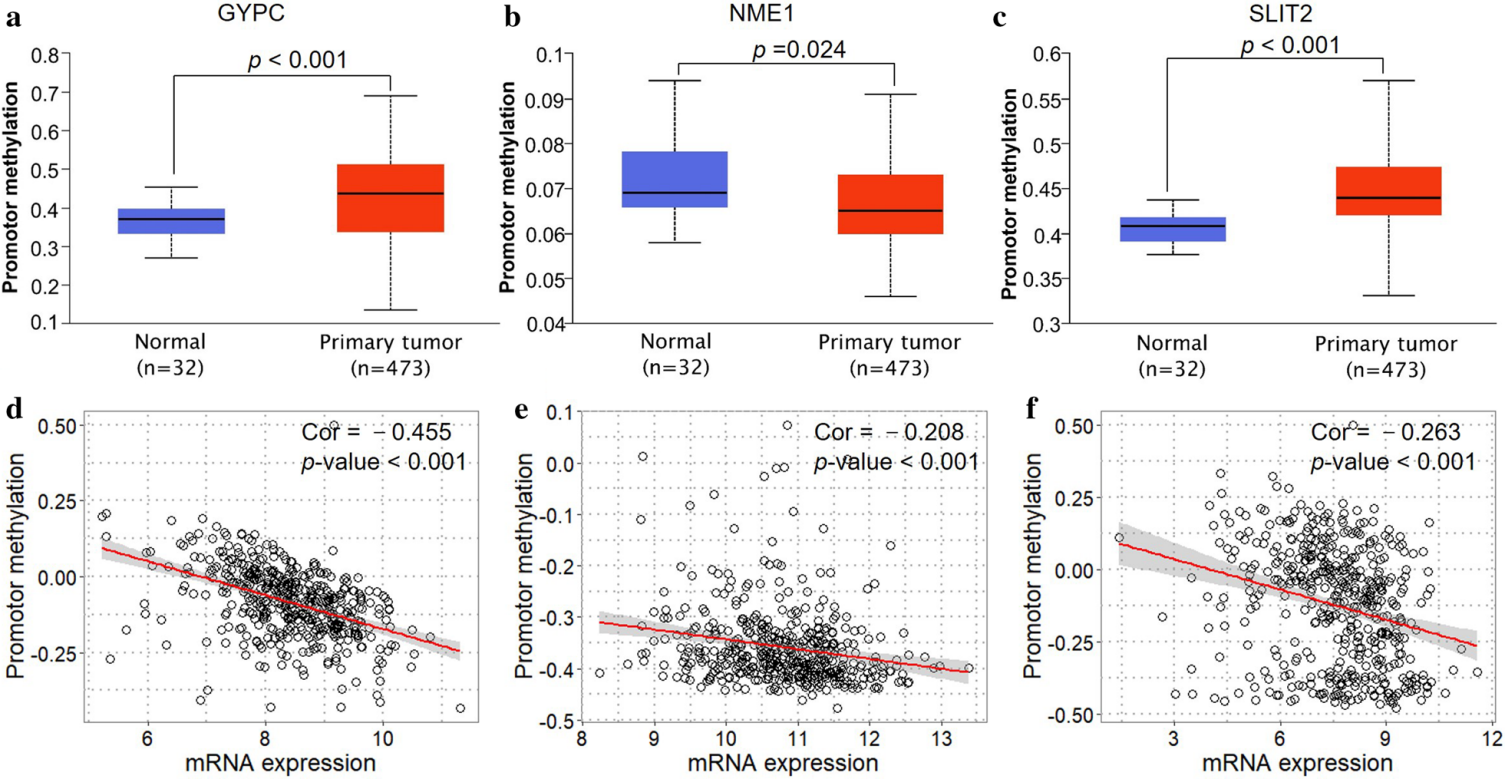
Correlation analysis between promoter methylation and mRNA expression levels in lung cancer. The promoter methylation levels of these three genes obtained from UALCAN online tool, including **GYPC** (**a**), NME1 (**b**), and SLIT2 (**c**). Pearson correlation analysis shows a significant positive correlation between gene expression and promoter methylation levels in LUAD, including GYPC (**d**), NME1 (**e**), and SLIT2 (**f**), the red line represents linear regression of data

**Fig. 10:**
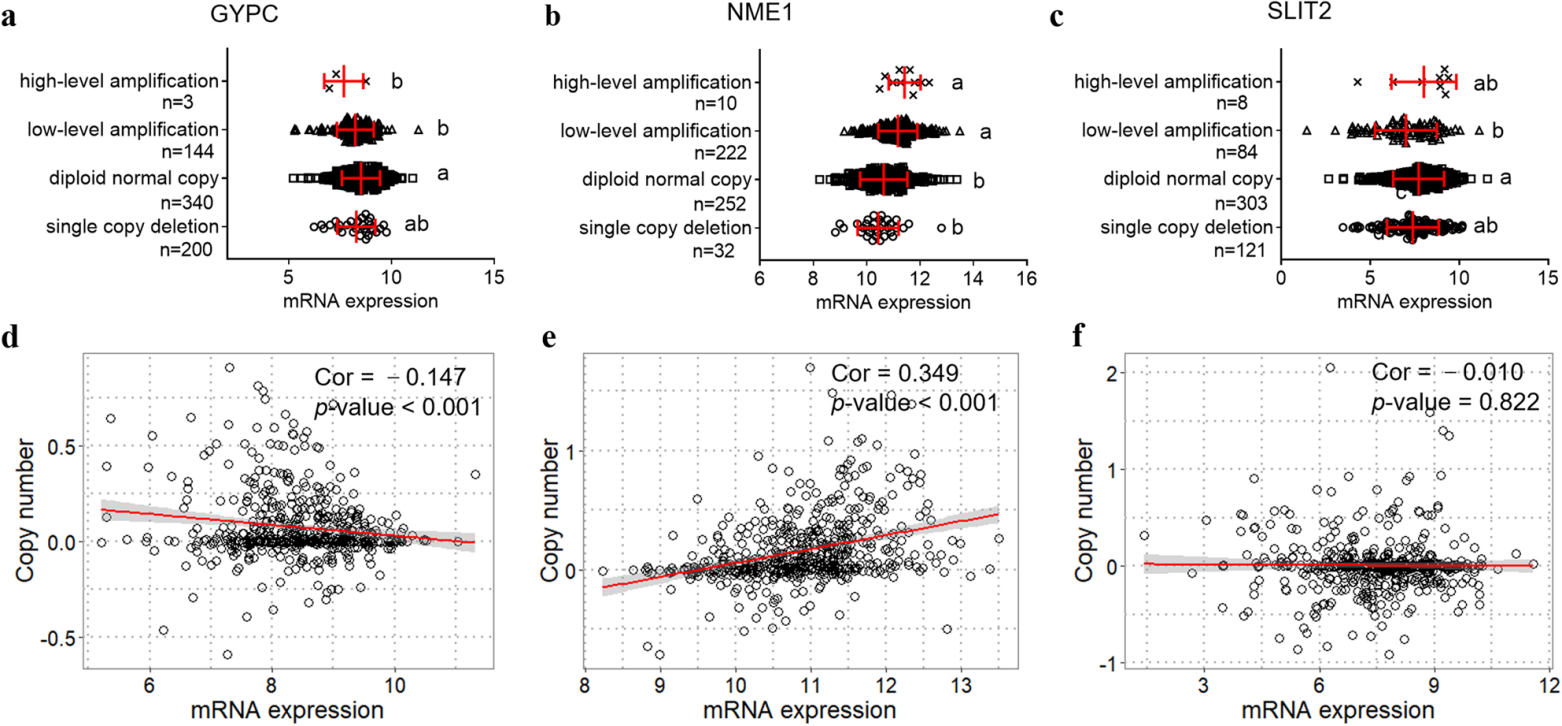
Correlation analysis between gene copy numbers and mRNA expression levels in lung cancer. Gene expression levels of GYPC (**a**), NME1 (**b**), and SLIT2 (**c**) in LUAD tissues with different amplification states. Pearson correlation analysis shows a significant positive correlation between gene expression levels and copy numbers in LUAD, including GYPC (**d**), NME1 (**e**), and SLIT2 (**f**), the red line represents linear regression of data

To understand possible regulation mechanisms of dysregulation of these three genes, we analyzed the public multi-omics datasets in the TCGA database. Based on the UALCAN online tool, the promoter regions of *GYPC* and *SLIT2* were found hyper-methylated significantly, as well as the *NME1* promoter was hypo-methylated (Fig. 9a–c). Also, further person correlation analysis suggested that these three genes mRNA expression levels were remarkedly negatively correlated with promoter methylation levels (*r* = − 0.455, − 0.208 and − 0.263, all *p* < 0.001, Fig. 9d–f). Besides, the up-regulated *NME1* expression was found significantly positively associated with increased gene amplification (*r* = 0.349, *p* < 0.001, Fig. 10b, e). Unexpectedly, the expression of *GYPC* was found negatively correlated to gene amplification (*r* = −0.147, *p* < 0.001, Fig. 10a, d). However, there is no significant correlation between *SLIT2* expression and gene amplification (*r* = −0.010, *p* = 0.822, (Fig. 10c, f).

## Discussion

Cigarette smoking is the primary risk factor for lung cancer development, and it is a significant contributor to the morbidity and mortality of LUAD patients [10, 11]. Recent studies have shown that histologically similar lung tumors have different molecular mechanisms of carcinogenesis because of different smoking status [12]. Thus, the mechanism of lung cancer in smokers and non-smokers needs to be assessed differently.

With the rapid development of sequencing and data analysis technologies, some essential genes related to LUAD have been identified through bioinformatics analysis. For example, *SPP1* has been identified as a prognostic biomarker in four LUAD datasets in the GEO database, which was also validated by the TCGA database [13]. Fan et al suggested 12 significant biomarkers that could distinguish lung cancer patients with different risks from the GEO database [14]. Gan et al identified the aberrantly expressed miR-375 gene involved in LUAD through the comparison of miRNA expression profiles in cancerous tissues based on the analysis and validation from TCGA and GEO datasets and published studies[15]. In the present study, three smoking-related signature genes, namely *GYPC*, *NME1* and *SLIT2*, were identified by an integrated analysis on the LUAD datasets in the GEO database and the high throughput sequencing data of cigarette smoke-induced malignant transformed BEAS-2B cells. In particular, *NME1* was reported increased by cigarette smoking in oral squamous cell carcinoma (OSCC) [16], and the lower expression of *SLIT2* were found in the lungs of cigarette smoke-induced emphysema mice [17].

Glycophorin C (*GYPC*) is an integral membrane glycoprotein. A recent study suggested *GYPC* can be used as a biomarker of breast cancer [18]. Increased *GYPC* gene expression was also reported to correlate with a worse outcome in childhood acute lymphoblastic leukemia [19]. However, there is limited evidence for the function of *GYPC* in the majority of solid tumors, especially in lung cancer. Our present study suggested that increased expression of *GYPC* was associated with a favorable outcome in LUAD patients. GSEA enrichment analysis indicated that *GYPC* was significantly associated with the JAK/STAT and cell adhesion signaling pathways which are essential to lung cancer progression and migration.

The NME/NM23 nucleoside diphosphate kinase 1 (NM23-H1, NME1) is a metastasis-associated gene the expression of which was correlated with various tumors. The expression level of *NME1* was found to be inversely proportional to the metastasis potential of several cancers, including breast cancer [20], gastric cancer [21], melanoma [22] and colon adenocarcinoma [23]. These results indicate that NME1 may act as a metastasis suppressor in these tumors. More importantly, elevated expression of NME1 has a better prognosis outcome in patients with breast cancer [24] and melanoma [25]. Instead, higher *NME1* expression was significantly associated with poor prognosis in patients with neuroblastoma [26] and osteosarcoma [27], as well as cervical cancer [28]. In this study, increased mRNA expression of *NME1* was found to be related to LUAD invasion depth and lymph node metastasis. It is noteworthy that decreased expression of *NME1* was associated with an improved prognosis in LUAD patients. Studies have shown that lymphatic metastasis is directly associated with distant recurrence and poor overall survival (OS) in non-small cell lung cancer patients [29]. We further found that *NME1* was involved in the cell cycle pathway, and the defect of cell cycle regulation has been reported to contribute to uncontrolled cancer cell proliferation [30]. Thus, we suggested that *NME1* plays a different role in LUAD than many other cancers, and it may serve as a potential biomarker for LUAD.

*SLIT2*, a secreted glycoprotein of the SLIT family [31], is involved in the epithelial-mesenchymal transition (EMT) process [32], which permits cancer cells to acquire migratory, invasive, and stem-like properties [33]. The knockdown of the *SLIT2* gene promoted the growth of gastric cancer cells and metastasis through activation of the AKT/$\beta$-catenin-mediated signaling pathway [34]. Another study demonstrated that decreased expression of *SLIT2* is associated with a poor prognosis and brain-specific metastasis in breast cancer patients [35]. The results in the present study showed that *SLIT2* was down-regulated in cigarette smoke-exposed cells and LUAD, and increased expression of *SLIT2* was associated with a better outcome in LUAD patients. Furthermore, *SLIT2* was significantly enriched in the TGF-$\beta$ signaling and focal adhesion pathways by enrichment analysis. Since both signaling pathways contribute to EMT activation, we suggested that *SLIT2* might serve as a potential tumor metastasis indicator.

The study of epigenetics provides an important clue for understanding the genesis and development of lung cancer [36]. Recent studies suggested that during the genesis of lung cancer, the promoter methylation levels of genes associated with multiple cellular functions are increased [37]. Our analysis determined that these genes expression levels were significantly negatively correlated with the promoter methylation status, which indicated that they may be gnomically regulated by DNA methylation. Besides, we analyzed the correlation between gene expression levels and copy numbers, which is another regulation mechanism at genome level. Copy number variation (CNV) is generally considered to be any genomic alteration greater than 50 base pairs in length [38], and it has been shown to play an essential role in human cancer. The loss of tumor suppressor genes and the gain of proto-oncogenes can contribute to cancer development [39, 40]. In this study, only *NME1* was found to have a significant positive correlation among expression and copy numbers, indicating that *NME1* was regulated not only by promoter methylation, but by copy numbers.

## Conclusions

In conclusion, our results indicated that *GYPC*, *NME1* and *SLIT2* may play a vital role in the development of smoking-related LUAD, which will be helpful in predicting the prognosis of LUAD patients. Mechanically, these three genes may be regulated by DNA methylation. Further in vitro and in vivo studies are needed to gain insights into the underlying molecular mechanisms of these three genes in LUAD.

## Methods

### BEAS-2B cell culture and cigarette smoke exposure

Human bronchial epithelial cells (BEAS-2B) and human LUAD cell lines (PC9, A549, H1975 and H1299) were purchased from the American Type Culture Collection (ATCC, USA). They were maintained in basal LHC-8 nutrient medium (Thermo Fisher Scientific, Waltham, MA, USA) or Dulbecco’s Modified Eagle Medium (DMEM, Invitrogen, MA, USA) in an incubator maintaining at 37 °C and humidified atmosphere of 5% CO$_{2}$. The *in vitro* model for malignant transformation was established by exposing BEAS-2B cells to cigarette smoke continuously for 5, 10, 20 and 30 passages (S5, S10, S20 and S30, separately), and this has been described in detail previously [41, 42].

### RNA isolation and high-throughput sequencing

Total RNA was isolated from normal BEAS-2B and S30 cells with TRIzol RNA isolation reagent (Invitrogen, MA, USA) according to the manufacturer’s protocol. Three biological replicates per group were used for mRNA sequencing analysis. A total of 1.5 $\mu$g RNA per sample was used as input material for the RNA library construction. The mRNA sequencing procedure has been described in detail in our previous article, and the raw data has been deposited in the Sequence Read Archive (SRA) database (https://trace.ncbi.nlm.nih.gov/Traces/sra/) with identifier SRP181756 [43]. Genes were computed by summing the fragments per kilo-base of exon per million fragments mapped (FPKM) of transcripts in each gene group.

### Human database extraction

Four datasets, GSE27262 [44], GSE19188 [45], GSE76760 [46] and GSE19804 [47], were downloaded from the Gene Expression Omnibus (GEO) database for differential expression analysis (Additional file 1: Table S1). All datasets met the following two criteria: (1) tissue samples obtained from human LUAD and adjacent normal tissues; and (2) each set included at least 50 samples. An additional six other datasets with survival information were downloaded for survival analysis (Additional file 1: Table S1). In these datasets, only LUAD and normal samples were retrieved and analyzed. The RNA-seq by the expected maximization (RSEM) data and the corresponding clinical information of LUAD in The Cancer Gene Atlas (TCGA) database were obtained from Xena (https://xena.ucsc.edu). The RSEM gene expression measurements for LUAD cases were transformed by using log$_{2}$ (RSEM + 1).

### Differential expression and integration analysis

For our RNA sequencing data, differential expression analysis of normal BEAS-2B and S30 cells was performed using the “DESeq2” R package [48]. For GEO and TCGA datasets, the “Limma” package was subsequently employed for identifying differentially expressed genes (DEGs) in each dataset [49]. |log2FC$|>$1 and a *p*-value < 0.05 were considered statistically significant for the DEGs. Gene integration for the DEGs identified from the four datasets was conducted using another R package “RobustRankAggreg” [50]. The expression levels of integrated genes in four GEO datasets were visualized with the R package “pheatmap” (https://cran.r-project.org/web/packages/pheatmap/index.html). Furthermore, a Venn diagram was generated by the “VennDiagram” R package (https://cran.r-project.org/web/packages/VennDiagram/index.html) to visualize the genes with the consistent change in S30 cells and LUAD samples.

### Real-time quantitative PCR

The total RNA of cells (including Beas-2B, S5, S10, S20 and S30 cells, as well as 4 LUAD cell lines) was isolated using TRIzol reagent (Invitrogen, MA, USA) according to the manufacturer’s protocol. A total amount of 1.5 $\mu$g of total RNA from each sample was reversely transcribed into complementary DNA (cDNA) using Revert Aid First Strand Complementary DNA Synthesis Kit (Thermo Fisher Scientific, Waltham, MA, USA) according to the manufacturer’s instructions. Quantitative PCR (qPCR) was performed using NovoScript$^{\textregistered }$SYBR Two-Step qRT-PCR Kit (novoprotein, China) on QuantStudioTM 6 Flex qRT-PCR system (Applied Biosystems, Foster City, CA, USA). GAPDH was used as a reference. The primer pairs used for qPCR in this study were listed in Table 3.

**Table 3:** Primers used in this study

Primer name	Primer sequence 5′ to 3′
F_GYPC	GCCGGATGGCAGAATGGAG
R_GYPC	GGAGGGAGACTAGGACGATGG
F_NME1	AAGGAGATCGGCTTGTGGTTT
R_NME1	CTGAGCACAGCTCGTGTAATC
F_SLIT2	AGCCGAGGTTCAAAAACGAGA
R_SLIT2	GGCAGTGCAAAACACTACAAGA
F_GAPDH	CTGGGCTACACTGAGCACC
R_GAPDH	AAGTGGTCGTTGAGGGCAATG

### Immunofluorescence analysis

The S30 and unexposed BEAS-2B cells were fixed in PBS containing 4.0% paraformaldehyde without methanol. The cells were washed and permeabilized with 0.2% Triton X-100 and blocked with 5% goat serum for 1 hour at room temperature. Diluted antibodies for human NME1 (11086-2-AP, Proteintech, Chicago, IL, USA), SLIT2 (20217-1-AP, Proteintech, Chicago, IL, USA) or GYPC (ab108619, Abcam, Cambridge, MA, USA) were added drop by drop and the slides were kept in a wet box at 4 °C overnight. Following incubated with FITC-conjugated goat anti-rabbit IgG for 1 hour at room temperature, the slides were washed, and the nuclei were counter-stained with 4,6-diamidino-2-phenylindole (DAPI). Photographs were taken and visualized using an FV1200MPE multiphoton laser scanning microscope (FV1200, OLYMPUS, Japan). The acquisition parameters were held constant for all the experiments.

### Western blot analysis

Total protein was extracted with RIPA buffer, and 20 μg of extracted total proteins were separated on SDS-PAGE gel and transferred onto a PVDF membrane (Millipore, Billerica, MA). After blocking with 5% bovine serum album (BSA, solarbio, China), the membrane was incubated at 4 °C overnight with various primary antibodies, including NME1, SLIT2 and GYPC. The HRP-labeled secondary antibody was used according to the host species of the primary antibody. Western blots were developed using electrochemiluminescence (ECL) substrate and visualized using the GeneTools GBox (Syngene, Frederick, MD, USA) system. The intensity of each spot was quantified using NIH ImageJ software (NIH, Bethesda, MD, USA).

### Analysis of patient smoking and pathological features

The mRNA expression levels of integrated genes in LUAD patients with different smoking histories were examined to identify the genes related to smoking. All samples in the TCGA LUAD and GEO datasets were divided into different groups according to the patient’s clinical features, including gender, age and invasion depth, as well as the status of lymph node metastasis, distant metastasis and tumor-node-metastasis (TNM) stage to find an association with mRNA expression levels.

### Enrichment analysis

To investigate the potential biological function of these three smoking-related genes, genes related to them were extracted from UALCAN (http://ualcan.path.uab.edu/index.html) with the Pearson coefficient $\ge$ 0.3 [51]. Gene Ontology (GO) and Kyoto Encyclopedia of Genes and Genomes (KEGG) pathway enrichment analysis were performed using the DAVID online tool (https://david.ncifcrf.gov/). Besides, the gene set enrichment analysis (GSEA, http://software.broadinstitute.org/gsea/index.jsp)[52] was used to verify the biological processes and KEGG pathways related to these genes. For the GSEA parameters, “1000”, “gene_set”, “weighted”, and “Pearson” were selected as “Number of permutations”, “Permutation type”, “Enrichment statistic”, and “Metric for ranking genes”, respectively.

### Survival analysis

The univariate Cox regression models were used to calculate the hazard ratios (HRs) and the 95% confidence intervals (CIs) based on *GYPC*, *NME1* and *SLIT2* expression levels in LUAD patients in six GEO datasets (GSE13213, GSE26939, GSE30219, GSE41271, GSE42127 and GSE14814) and the TCGA LUAD dataset. The Cox analysis was performed using the “survival” (https://cran.r-project.org/web/packages/survival/index.html) package, and the “survminer” (https://github.com/kassambara/survminer) package was used to generate Kaplan-Meier (KM) survival curves. All LUAD patients were divided into high and low expression groups according to the median values of mRNA expression level.

### Copy number and methylation analysis

To further understand the regulatory mechanisms of these three genes, we used the multi-omics data in the TCGA database for further correlation analysis. The promoter methylation levels of these three genes in TCGA LUAD dataset were performed using UALCAN . Besides, the methylation and copy number variation (CNV) data in the TCGA LUAD dataset was obtained from LinkedOmics (http://www.linkedomics.orglogin.php), and Pearson coefficient was calculated to demonstrate the relationship between them and mRNA expression levels.

### Statistical analysis

In this study, heat map, survival, and differential expression analyses were performed with the R software packages. Experimental data were analyzed using GraphPad Prism 7 (GraphPad Software Inc., La Jolla, CA, USA). Data were reported as the mean ± standard deviation (SD) of three independent experiments. Data were analyzed using Student’s t-test to compare between two groups. *p*-values < 0.05 were considered significant.

## Supplementary information

**Additional file 1.** Additional tables.

## Data Availability

The mRNA sequencing dataset generated for this study can be found in the Sequence Read Archive (SRA) database (https://trace.ncbi.nlm.nih.gov/Traces/sra/) with identifier SRP181756. The GEO datasets analyzed for this study can be obtained from the GEO database (https://www.ncbi.nlm.nih.gov/geo/query/acc.cgi) with corresponding identifier as list in Additional file 1: Table S1. The TCGA LUAD dataset analyzed for this study can be obtained from UCSC Xena (https://xenabrowser.net/datapages/).

## Abbreviations

LUAD: Lung adenocarcinoma
BEAS-2B: Normal lung human bronchial epithelial cells
S30: Cells exposed to cigarette smoke continuously for 30 passages
DEGs: Differentially expressed genes
RRA: RobustRankAggreg
GEO: Gene Expression Omnibus
TCGA: The Cancer Gene Analysis
GSEA: Gene Set Enrichment Analysis
qPCR: Quantitative PCR
IF: Immunofluorescence
WB: Western blot
GYPC: Glycophorin C
NME1: NME/NM23 nucleoside diphosphate kinase 1
SLIT2: Slit guidance ligand 2
OS: Overall survival
ATCC: American Type Culture Collection
DMEM: Dulbecco’s Modified Eagle Medium
RSEM: RNA-seq by the expected maximization
TNM: Tumor-node-metastasis
TNM: Tumor-node-metastasis
CIs: Confidence intervals
KM: Kaplan–Meier
MSigDB: Molecular Signatures Database
cDNA: Complementary DNA
DAPI: 4,6-diamidino-2-phenylindole
SD: Standard deviation
EMT: Epithelial–mesenchymal transition
CNV: Copy number variation
