# Supplementary material for: Identification and validation of smoking-related genes in lung adenocarcinoma using an in vitro carcinogenesis model and bioinformatics analysis
Source: J Transl Med. 2020 Aug 14;18:313. doi: 10.1186/s12967-020-02474-x (PMC7427766; doi:10.1186/s12967-020-02474-x)
Supplement: Supplementary file 1 — Additional file 1. Additional tables. [file 12967_2020_2474_MOESM1_ESM.pdf]

Additional file 1 — Supplementary Table S1 Data cohort characteristics

Data set	Use	LUAD (N)	Normal (N)
GSE27262	Integration Analysis	25	25
GSE19188	Integration Analysis	45	65
GSE76760	Integration Analysis	27	27
GSE19804	Integration Analysis	60	60
GSE13213	Cox/KM analysis, Smoking history	117	0
GSE26939	Cox/KM analysis, Smoking history	115	0
GSE41271	Cox/KM analysis, Smoking history	185	0
GSE30219	Cox/KM analysis	85	0
GSE42127	Cox/KM analysis	133	0
GSE14814	Cox/KM analysis	71	0
TCGA	Cox/KM analysis, Smoking history, Pathological	515	59

LUAD: Lung adenocarcinoma; TCGA: The Cancer Gene Atlas; KM: Kaplan-Meier.

Additional file 2 — Supplementary Table S2 Gene integration for the DEGs identified from the four datasets

Change	Genes symbol
Down	AGER, CA4, FAM107A, CLEC3B, EXOSC7, GPM6A, KANK3, TNNC1, FABP4, FAM189A2, TEK, EDNRB, PTPRB, CDH5, ADAMTSL3, FOXF1, TCF21, AKAP2, PALM2-AKAP2, RGCC, HIGD1B, FCN3, FIGF, PIR-FIGF, EMP2, JAM2, IGSF10, CAV1, SPOCK2, SASH1, GRK5, SGCG, LIMS2, MMRN2, ADAMTS8, ACADL, KCNK3, ADIRF, RASIP1, SEMA5A, SLC6A4, TGFBR3, FXYD1, CD93, KLF2, AOC3, ARHGAP6, EMCN, PECAM1, TNXA, TNXB, CLIC5, DENND3, MYZAP, FHL1, C14orf132, CFD, LDB2, PIP5K1B, LYVE1, RAMP2, AGTR1, VIPR1, TMEM100, ADRB2, ARRB1, CASKIN2, ITIH5, PCAT19, SLIT2, SLIT3, SEMA3G, S1PR1, ADH1B, STX11, SYNM, GNG11, KIAA1462, TRHDE, HEG1, RAMP3, HSPB8, LINC00312, LINC00968, DPYSL2, GIMAP1-GIMAP5, GIMAP5, CD36, FGR, SELP, ACVRL1, LOC101927458, LPHN2, HBA1, HBA2, SH3BP5, KAL1, HBB, CBFA2T3, GIMAP6, HYAL1, MFAP4, ASPA, P2RY14, AOX1, ANGPT1, WIF1, COX7A1, THBD, WFS1, GPIHBP1, LRRC32, SDPR, WISP2, LIMCH1, LHFP, GDF10, SPARCL1, RTKN2, GPM6B, MARCO, LINC00341, SYNE3, MIR22, MIR22HG, MYH10, LAMP3, ROBO4, ICAM2, RASL12, DAPK2, MYCT1, KLF4, CLDN18, PAPSS2, CD97, PPAP2B, STXBP6, ACKR4, GMFG, CCDC68, S100A4, MT1M, GRIA1, PALMD, MME, CBX7, CAV2, PCOLCE2, ECSCR, CDO1, OR7E47P, CRIM1, LOC101929500, DPEP2, NEDD4L, LPL, SOX17, KL, GHR, FMO2, CALCRL, VSIG4, SOCS2, GADD45B, PTRF, VWF, FBLN5, CLIC3, GYPC, MIR6872, SEMA3B, CACNA2D2, LRRN3, CHRDL1, PPP1R15A, BMP2, SFTPC, IL33, IL1RL1, SRPX, CFP, FGF2, TMEM47, MAL, OLFML1, AKAP12, CLDN5, PPBP, ANXA3, FOSB, C5AR1, PLLP, ITM2A, PKIG, NDNF, VGLL3, CCDC85A, KLRF1, GIMAP4, RASSF2, PDE2A, CD52, DUSP1, LIFR, TBX2, ZBED2, IL18R1, GPX3
Up	SPP1, PYCR1, GGCT, CDH3, COL10A1, ZWINT, GALNT7, TOP2A, NME1, HMGB3, KIF20A, FUT2, SRD5A1, OCIAD2, UBE2C, KIAA0101, CRABP2, STK39, LAD1, RRM2, CCNB1, FUT3, TOX3, PRDX4, MELK

Additional file 3 —Supplementary Table S3 Association of mRNA expression with LUAD patient tobacco smoking history in the TCGA database

Smoking history	1 (N=75)	2 (N=119)	3 (N=135)	4 (N=168)
S1PR1	8.74±0.97	8.44±1.00*	8.82±0.89##	8.75±0.94##
CRABP2	10.42±2.36	10.34±2.63	10.42±2.44	10.70±2.32
PTPRB	9.40±1.02	9.08±1.14*	9.36±1.02#	9.31±1.05
KIAA1462	9.83±0.95	9.51±1.11*	9.58±0.87	9.64±1.00
SRPX	7.10±1.16	6.68±1.45*	6.94±1.18	6.90±1.19
MME	7.70±1.66	7.51±2.02	7.13±1.64*	7.40±1.76
GYPC	8.64±0.93	8.20±1.00**	8.39±0.82	8.47±0.92#
THBD	9.34±1.03	9.15±1.16	9.63±1.12##	9.51±1.11##
NME1	10.64±0.98	11.06±0.70**	10.62±0.91##	10.99±0.80**
SEMA3B	8.64±1.58	8.48±1.98	8.90±1.61	8.60±1.74
SLIT2	7.85±1.43	7.17±1.53**	7.69±1.50##	7.54±1.55#
LIMCH1	11.27±0.84	11.07±1.32	11.41±0.96#	11.25±1.04
SYNE3	4.84±1.12	4.61±1.35	4.53±1.32	4.65±1.11

Note: 1=Lifelong Non-smokers (less than 100 cigarettes smoked in Lifetime), 2=Current smokers (includes daily smokers and non-daily smokers or occasional smokers), 3=Current reformed smokers for >15 years (greater than 15 years), 4=Current reformed smokers for ≤15 years (less than or equal to 15 years). Data represented are Mean±SD, n depend on how many valid LUAD samples with corresponding factors. SD indicates standard deviation. *$p<0.05$, versus Lifelong Non-smokers; **$p<0.01$, versus Current smokers; #$p<0.05$, versus Lifelong Non-smokers; ##$p<0.01$, versus Current smokers.
